# Evaluation of Anticancer Therapy‐Related Tumor Flare Reaction: Insights From Food and Drug Administration's Adverse Event Reporting System Dataset

**DOI:** 10.1002/cam4.71660

**Published:** 2026-03-09

**Authors:** Wei Wang, Jiayuan Cui, Yuxuan Bo, Yuxuan Wang, Zhipeng Sun, Xin Guan, Baoan Hong, Ning Zhang

**Affiliations:** ^1^ Department of Urology, Beijing Anzhen Hospital Capital Medical University Beijing P. R. China; ^2^ Department of Nuclear Medicine, First Hospital of Shanxi Medical University Shanxi Medical University Taiyuan Shanxi P. R. China

**Keywords:** adverse events, anticancer drugs, FAERS, tumor flare reaction

## Abstract

**Aim:**

Tumor flare reaction (TFR) is characterized by an increase in tumor size during immunotherapy, often resembling disease progression. This study explores the relationship between anti‐tumor drugs and tumor flare reaction (TFR) through the FAERS database to assist clinicians in better patient management.

**Methods:**

We analyzed the FAERS database to identify TFR cases reported from Q1 2004 to Q3 2024. For signal detection, we employed disproportionality analysis with four algorithms—reported odds ratio (ROR), proportional reporting ratio (PRR), Bayesian confidence propagation neural network (BCPNN), and empirical Bayes geometric mean (EBGM). These algorithms assessed statistical correlations between anticancer drugs and TFR, based on a 2 × 2 contingency table framework.

**Results:**

From Q1 2004 to Q3 2024, 566 TFR cases were recorded in the FAERS database. The incidence of TFR peaked in 2023, with the highest increase in cases from 2021 to 2022, at 4.9%. A total of 28 anticancer drugs were identified as strongly associated with TFR, of which only 4 are explicitly listed in the medication instructions as having TFR‐related adverse reactions. Lenalidomide was the most frequent drug causing TFR, accounting for 38% of all TFR reports.

**Conclusions:**

Our findings highlight the key associations between treatment drugs and TFR, particularly targeted therapies such as Rituximab, which are not explicitly marked for this side effect in the FAERS database. The study emphasizes the need for clinicians to closely monitor TFR in patients receiving certain cancer treatments and improve therapeutic strategies to mitigate TFR risks, ensuring safer cancer treatment outcomes.

## 1 Introduction

According to the National Cancer Institute Common Terminology Criteria for Adverse Events (NCI‐CTCAE v3.0), tumor flare reaction (TFR) is defined as a constellation of symptoms and signs occurring shortly after the initiation of therapy, including anti‐estrogens, androgens, or other hormonal agents [1]. Clinical manifestations include tumor pain, visible tumor inflammation, hypercalcemia, diffuse bone pain, and other electrolyte disturbances [1]. Although TFR can sometimes be transient and self‐limiting, its presentation often mimics true disease progression, which may lead to premature treatment discontinuation or unnecessary changes in therapy [2, 3].

Over the past decade, TFR has been increasingly recognized in hematologic malignancies, especially chronic lymphocytic leukemia (CLL), during treatment with immunomodulatory drugs (IMiDs) such as thalidomide and lenalidomide [4, 5, 6]. In CLL, TFR typically manifests as painful lymphadenopathy, sometimes accompanied by splenomegaly, fever, rash, or transient lymphocytosis [4, 5]. Similar flare phenomena have also been reported with immune checkpoint inhibitors in solid tumors [3, 7]. In this context, radiographic findings such as new lesions or an apparent increase in tumor burden may resemble disease progression, but may actually reflect immune‐cell infiltration and the subsequent development of antitumor immune responses [3]. The American Society of Clinical Oncology (ASCO) and the American Society of Hematology (ASH) have noted that the incidence of TFR in CLL ranges between 28% and 58%, making it a relatively frequent treatment‐related event [8].

A critical distinction, however, must be made between TFR and pseudoprogression. Both conditions may present with transient increases in tumor burden, but their mechanisms and clinical features differ. TFR is primarily an acute inflammatory response closely linked to therapy initiation and is often accompanied by systemic symptoms such as pain, fever, or rash. In contrast, pseudoprogression refers to radiological evidence of tumor enlargement or new lesions during immunotherapy, followed by subsequent regression without treatment modification [8]. Clinically, TFR tends to occur early and is symptomatic, whereas pseudoprogression is usually asymptomatic and recognized mainly on serial imaging. Because these two phenomena are easily misdiagnosed as true progression, careful differentiation is essential in both research and practice, as inappropriate attribution could lead to premature treatment discontinuation or misjudgment of therapeutic efficacy [8].

Before the establishment of large‐scale pharmacovigilance resources, studies on the association between anticancer drugs and TFR were largely limited to case reports and small clinical series [4, 5]. This limited the ability to generalize across tumor types or drug classes. The US Food and Drug Administration's Adverse Event Reporting System (FAERS) provides a valuable real‐world dataset that systematically collects reports of adverse drug reactions, including those related to conventional and novel anticancer therapies. Although FAERS data cannot establish incidence or causality, they can generate important early‐warning signals and help raise clinical awareness.

Accordingly, the present study used FAERS data from Q1 2004 to Q3 2024 to investigate reports of TFR across anticancer therapies. Rather than providing precise epidemiologic estimates, our aim was to highlight potential associations, enhance clinical recognition, and emphasize that apparent progression in some cases may represent a flare reaction rather than treatment failure. By doing so, we hope to provide clinicians with a pragmatic reference point for decision‐making and to underscore the importance of cautious interpretation of early tumor changes in oncology practice.

## 2 Methods

### 2.1 Study Design, Data Source, and Preprocessing

This study was a cross‐sectional pharmacovigilance analysis based on the US Food and Drug Administration (FDA) Adverse Event Reporting System (FAERS), a global spontaneous reporting system that collects adverse event (AE) and medication error reports associated with drugs and biologics from healthcare professionals, manufacturers, and consumers. We retrieved all reports from Q1 2004 to Q3 2024. Data extraction and preprocessing were conducted using R version 4.4.3 (R Foundation for Statistical Computing, Vienna, Austria). Following FDA recommendations, duplicate reports were consolidated by CASEID and version/date (retaining the most recent record), and records flagged as duplicates were removed. Drug names were standardized to RxNorm, and adverse events were mapped to MedDRA terminology (version 27.0) to ensure consistency and accuracy. Because FAERS is a spontaneous reporting system, detailed treatment regimens, biomarker tests, clinicopathological variables, and biochemical assays are not systematically captured and were therefore not analyzed in this study.

### 2.2 Identification of Target AE Reports and Eligibility Criteria

In FAERS, AEs are coded using MedDRA's hierarchical terminology, with the Preferred Term (PT) as the second‐most granular level. Reports were included if they (i) involved FDA‐approved anticancer drugs and (ii) were coded with the PT “Tumor flare” (corresponding to the clinical construct of tumor flare reaction, TFR). Reports with missing essential drug identifiers or incomplete demographic information were excluded. When available, we extracted age, sex, reporting country, year of report, and reporter type. FAERS also specifies the role of the drug as primary suspect (PS), secondary suspect (SS), concomitant (C), or interacting (I); the present analysis focused on suspect drugs (PS/SS). The final analytic dataset contained *n* = 566 reports of TFR during the study period.

### 2.3 Disproportionality Analysis (Signal Detection)

Disproportionality analysis is a type of data mining algorithm designed to identify ADR signals quantitatively in sizable pharmacovigilance databases, helping to examine the variations in the background and occurrence frequencies for the target drug and target AEs, and thus proving a drug's statistical correlation with an AE. This study investigated the relationship between the frequency of TFR‐related AE occurrences for different drugs in the FAERS database. To improve the robustness of the results, we employed four algorithms: reported odds ratio (ROR), proportional reported odds ratio (PRR), bayesian confidence propagation neural network (BCPNN), and the multiple Gamma Poisson reduction method (EBGM). The construction of the four algorithms was based on the 2 × 2 table calculation principle (Table 1). ‘*a*’ denotes the number of individuals experiencing the targeted AE after administration of a specific anti‐cancer drug, ‘*b*’ indicates the occurrence of the other AEs in the presence of specific anti‐cancer drug, ‘*c*’ represents instances of targeted AE following administration of other drugs, and ‘*d*’ pertains to occurrences of other AEs in the presence of other drugs. The total population (*N*) is calculated using the formula *N* = *a* + *b* + *c* + *d*.

**TABLE 1:** Table matrix.

	Report of report of tumor flare reaction	Other adverse effects
Anti‐cancer drug	*a*	*b*
Other drugs	*c*	*d*
*N* = *a* + *b* + *c* + *d*		

Four widely used signal detection algorithms were applied:

- Reporting odds ratios (ROR) algorithm

$$\mathrm{ROR}=\left(\mathrm{ad}\right)/\left(\mathrm{bc}\right)$$

$$95\%\mathrm{CI}=e^{\ln\left(\mathrm{ROR}\right)\pm 1.96\sqrt{\left(\frac{1}{a}+\frac{1}{b}+\frac{1}{c}+\frac{1}{d}\right)}}$$

The criteria of positive safety signal detection: the lower limit of 95% CI > 1, *N* ≥ 3;

ii Proportional reporting ratios (PRR) algorithm

$$\mathrm{PRR}=\left[a\left(c+d\right)\right]/\left[c\left(a+b\right)\right]$$

$$x^{2}=\frac{\left(a+b+c+d\right){\left(\mathrm{ad}-\mathrm{bc}\right)}^{2}}{\left(a+b\right)\left(c+d\right)\left(a+c\right)\left(b+d\right)}$$

The criteria of positive safety signal detection: PRR ≥ 2, *χ* ^2^ ≥ 4, *N* ≥ 3;

iii BPCNN algorithm

$$\mathrm{IC}=\log_{2}\frac{a\left(a+b+c+d\right)}{\left(a+b\right)\left(a+c\right)}$$

$$95\%\mathrm{CI}=E\left(\mathrm{IC}\right)\pm 2\times \sqrt{V\left(\mathrm{IC}\right)}$$

$$V\left(\mathrm{IC}\right)=\frac{1}{{\left(\ln2\right)}^{2}}\left\{\begin{array}{l} \left[\frac{\left(a+b+c+d\right)-a+\gamma -\gamma 11}{\left(a+\gamma 11\right)\left(1+a+b+c+d+\gamma \right)}\right]\\ +\left[\frac{\left(a+b+c+d\right)-\left(a+b\right)+\alpha -\alpha 1}{\left(a+b+\alpha 1\right)\left(1+a+b+c+d+\alpha \right)}\right]\\ +\left[\frac{\left(a+b+c+d\right)-\left(a+c\right)+\beta -\beta 1}{\left(a+b+\beta 1\right)\left(1+a+b+c+d+\beta \right)}\right] \end{array}\right\}$$

$$\mathrm{IC}=\log_{2}\frac{p\left(x,y\right)}{p\left(x\right)p\left(y\right)}=\log_{2}\frac{a\left(a+b+c+d\right)}{\left(a+b\right)\left(a+c\right)}$$

$$E\left(\mathrm{IC}\right)=\log_{2}\frac{\left(a+\gamma 11\right)\left(a+b+c+d+\alpha \right)\left(a+b+c+d+\beta \right)}{\left(a+b+c+d+\gamma \right)\left(a+b+\alpha 1\right)\left(a+c+\beta 1\right)}$$

$$\gamma =\gamma 11\frac{\left(a+b+c+d+\alpha \right)\left(a+b+c+d+\beta \right)}{\left(a+b+\alpha 1\right)\left(a+c+\beta 1\right)}$$

$$\mathrm{IC}-2\mathrm{SD}=E\left(\mathrm{IC}\right)-2\sqrt{V\left(\mathrm{IC}\right)}$$

α1=β1=1;α=β=2;γ11=1

The criteria of positive safety signal detection: (IC – 2SD) > 0;

iv Empirical Bayes Geometric Mean (EBGM) algorithm

$$\text{EBGM}=\left(\text{aN}\right)/\left[\left(a+b\right)\left(a+c\right)\right]$$

$$95\%\mathrm{CI}=e^{\ln\left(\text{EBGM}\right)\pm 1.96\sqrt{\left(\frac{1}{a}+\frac{1}{b}+\frac{1}{c}+\frac{1}{d}\right)}}$$

The criteria of positive safety signal detection: EBGM05 > 2 (EBGM05: the lower bound of 95% CI).

It should be emphasized that disproportionality analyses based on spontaneous reporting systems such as FAERS cannot provide incidence rates or establish causal relationships. Instead, these measures indicate signals of disproportionate reporting that may reflect potential associations warranting further clinical or epidemiological investigation.

### 2.4 Study Endpoints

The primary endpoint was the detection of disproportionality signals between anticancer drugs and TFR. The secondary endpoints were: temporal distribution of TFR reports (2004Q1–2024Q3), demographic features (age, sex, reporting country, year, reporter type), and distribution of reports by anticancer drug class.

### 2.5 Statistical Analysis

All analyses were conducted using R version 4.4.3. Disproportionality metrics (ROR, PRR, BCPNN, EBGM) were computed using base R functions and validated pharmacovigilance packages (e.g., PhViD, openEBGM). Categorical variables (sex, reporter type, reporting country) were summarized as counts and percentages; continuous variables (age) were summarized as medians with interquartile ranges (IQR). No imputation was performed for missing data; such cases were categorized as “unknown.” Signal detection thresholds followed established pharmacovigilance criteria as specified above.

### 2.6 Ethics Statement

This study was exempt from Institutional Review Board approval because it used publicly available, de‐identified data from the FAERS database.

## 3 Results

### 3.1 Descriptive Analysis

**FIGURE 1:**
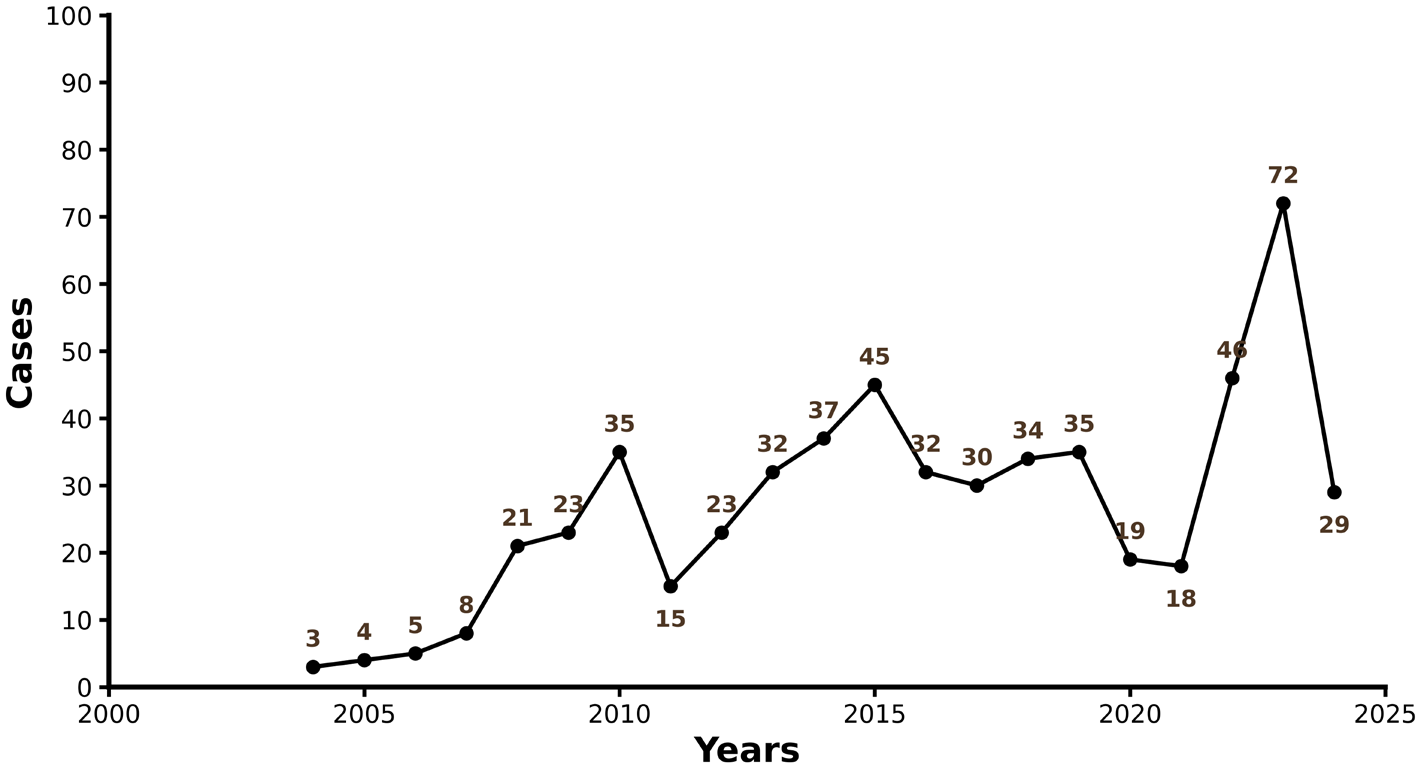
Trends in incidence of reported tumor flare reaction (TFR).

From Q1 2004 to Q3 2024, the FAERS database recorded 566 TFR cases. A small number of cases had missing baseline information, which was categorized as missing. The clinical characteristics of the patients are shown in Table 2. As shown in Figure 1, the reported incidence of TFR peaked in 2023, with 72 cases. Since 2008, the number of reported cases has fluctuated, but there has been an overall increase. The majority of affected individuals were male (250 cases, 44.2%), while female cases accounted for 116 individuals, or 29.3%. The average age was 60.4 ± 14.9 years, with a median age of 62.0 years (range: 0.6–91.0 years) (Table 2). Among the countries with a higher number of reports, the United States had the most (45.8%), followed by the United Kingdom (6.5%), France (4.8%), and Canada (3.5%) (Table 3). Based on the frequency of TFR cases, the top 10 cancer types are summarized in Table 4. The most common type was chronic lymphocytic leukemia (20.7%), followed by the use of unspecified drugs (10.4%), diffuse large B‐cell lymphoma (5.3%), and mantle cell lymphoma (4.9%). Among the top 10 cancer types, lymphomas accounted for 9, making up 47.2%. The most common solid tumor was prostate cancer, accounting for 2.1%.

**TABLE 2:** Clinical characteristics of reported tumor flare reaction.

Characteristics	Number
Sex	
Female	116 (29.3%)
Male	250 (44.2%)
Unknown	150 (26.5%)
Age	
Mean	60.4 (14.9)
Median	62.0 [0.600, 91.0]
Missing	256 (45.2%)
Total	*N* = 566

**TABLE 3:** The country information on reported TFR cases (Top 10).

Country	Cases
United States	256
Great Britain	37
France	31
Canadian	20
Japan	19
España	18
Italy	17
Bundesrepublik Deutschland	16
Country not specified	12
United Kingdom	11
Austria	8
Missing	8
Sweden	8
Commonwealth of Australia	7
Czech Republic	7

**TABLE 4:** The top 10 cancer types of reported tumor flare reaction.

Cancer type	Cases	Percentage
Chronic lymphocytic leukemia	117	20.70%
Product used for unknown indication	59	10.40%
Diffuse large B‐cell lymphoma	30	5.30%
Mantle cell lymphoma	28	4.90%
Follicular lymphoma	24	4.20%
Lymphoma	18	3.20%
Non‐Hodgkin's lymphoma	15	2.70%
B‐cell lymphoma	14	2.50%
Plasma cell myeloma	14	2.50%
Prostate cancer	12	2.10%

### 3.2 Description Based on Drugs

**FIGURE 2:**
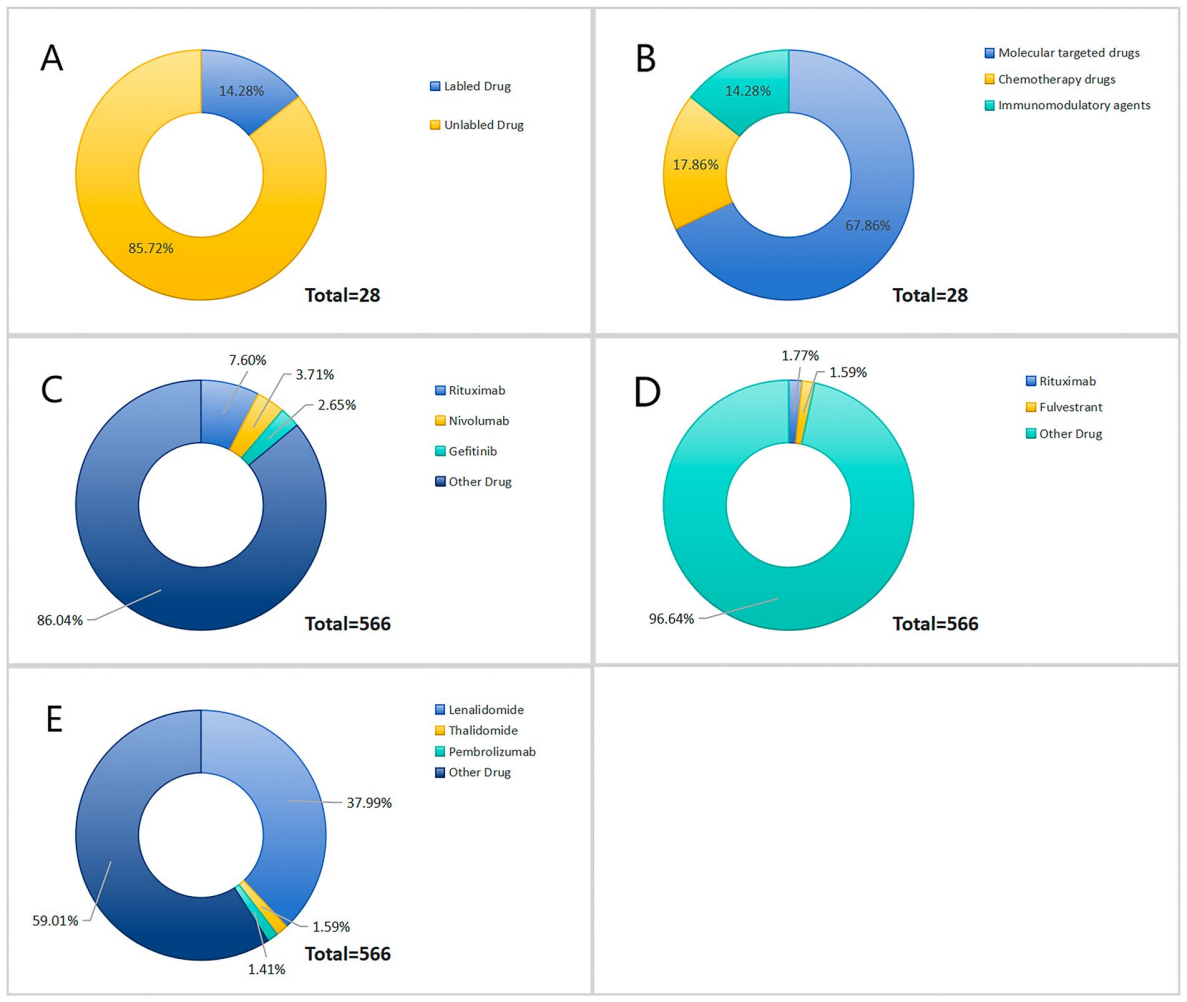
Proportional analysis of drug types. (A) 28 drugs were explicitly labeled as having TFR‐related adverse reactions in the FDA database, 4 drugs were not specifically labeled. (B) The total 28 drugs were categorized by their mechanisms of action into molecularly targeted drugs (67.86%), chemotherapy drugs (17.86%), Immunomodulatory agents (14.28%). (C) Among molecular targeted drugs, Rituximab accounts for 7.60% cases of all TFR‐related cases, followed by Nivolumab (21 cases, 3.71%) and Gefitinib (15 cases, 2.65%). (D) Among chemotherapy drugs, Bendamustine was the most reported drug with 10 (1.77%) cases among 566 TFR cases, followed by Fulvestrant (9 cases, 1.59%). (E) Among immunomodulatory agents, the most reported drug was Lenalidomide (37.99%), followed by Rituximab (7.60%), and Nivolumab (3.71%).

Using four statistical models (ROR, PRR, BPCNN, EBGM), we identified 28 anticancer drugs highly associated with TFR. Of these, only 4 drugs are explicitly labeled in the drug guidelines for TFR‐related adverse reactions; the remaining drugs were statistically related to TFR but not explicitly labeled in the FDA data (Figure 2A). The 28 drugs were categorized based on their mechanisms of action into molecular targeted drugs, chemotherapy drugs, and immunomodulatory agents (Figure 2B). In terms of drug categories, molecular targeted drugs accounted for the majority of TFR‐related reports, comprising 67.86% of all reports in the study. Chemotherapy drugs were the second most reported, accounting for 17.86% of all reports, with the fewest reports for immunomodulatory agents (14.28%) (Figure 2B). Among molecularly targeted drugs, Rituximab was the most reported, with 43 cases (7.60%) out of 566 TFR‐related reports, followed by Nivolumab (21 cases, 3.71%) and Gefitinib (15 cases, 2.65%) (Figure 2c). Among chemotherapy drugs, Bendamustine was the most reported, with 10 TFR‐related reports (1.77%), followed by Fulvestrant (9 cases, 1.59%) (Figure 2D). Among immunomodulatory agents, Lenalidomide was the most reported drug, accounting for 215 cases (37.99%) out of 566 TFR‐related cases, followed by Thalidomide (1.59%) and Pembrolizumab (1.41%) (Figure 2E). Of all 28 drugs, Lenalidomide had the most reports of TFR‐related adverse reactions to the FDA, accounting for 37.99% of all TFR reports, with the FDA drug guidelines explicitly indicating this side effect. However, Rituximab (7.60%) and Nivolumab (3.71%), the second and third most reported drugs, are not mentioned in the FDA's drug guidelines for TFR‐related adverse reactions.

### 3.3 Disproportional Analysis

As mentioned earlier, four statistical models were used in this study. Table 1 lists the ranking of 28 TFR‐related reports associated with anticancer drug use based on the four models that showed significance, excluding a few reports.

**FIGURE 3:**
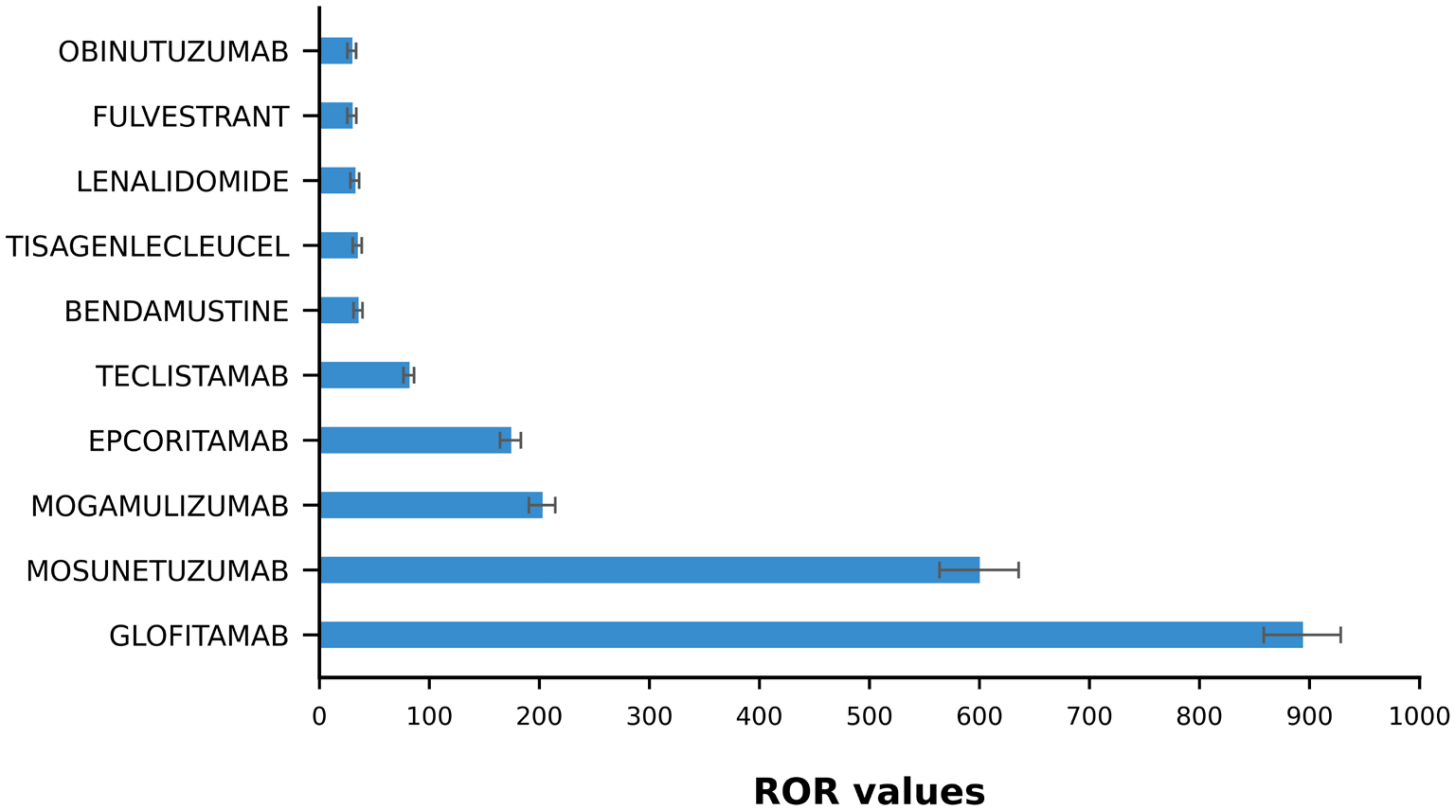
Top 10 drugs ranked by ROR values.

Specifically, we identified anticancer drugs significantly associated with TFR through ROR screening, with the lowest significant ROR being 5.55, and 50% of the associations showing an ROR greater than 17. Glofitamab had the highest ROR value at 893.29, followed by Mosunetuzumab (599.63) and Mogamulizumab (202.34). These three drugs are all used for molecular targeted therapies, primarily indicated for lymphocytic lymphoma. Only Glofitamab is explicitly labeled in the medication instructions as causing TFR‐related adverse reactions, suggesting that clinicians should be aware of the potential risk of TFR when using molecular‐targeted drugs in the treatment of lymphoma and related diseases (Figure 3).

**FIGURE 4:**
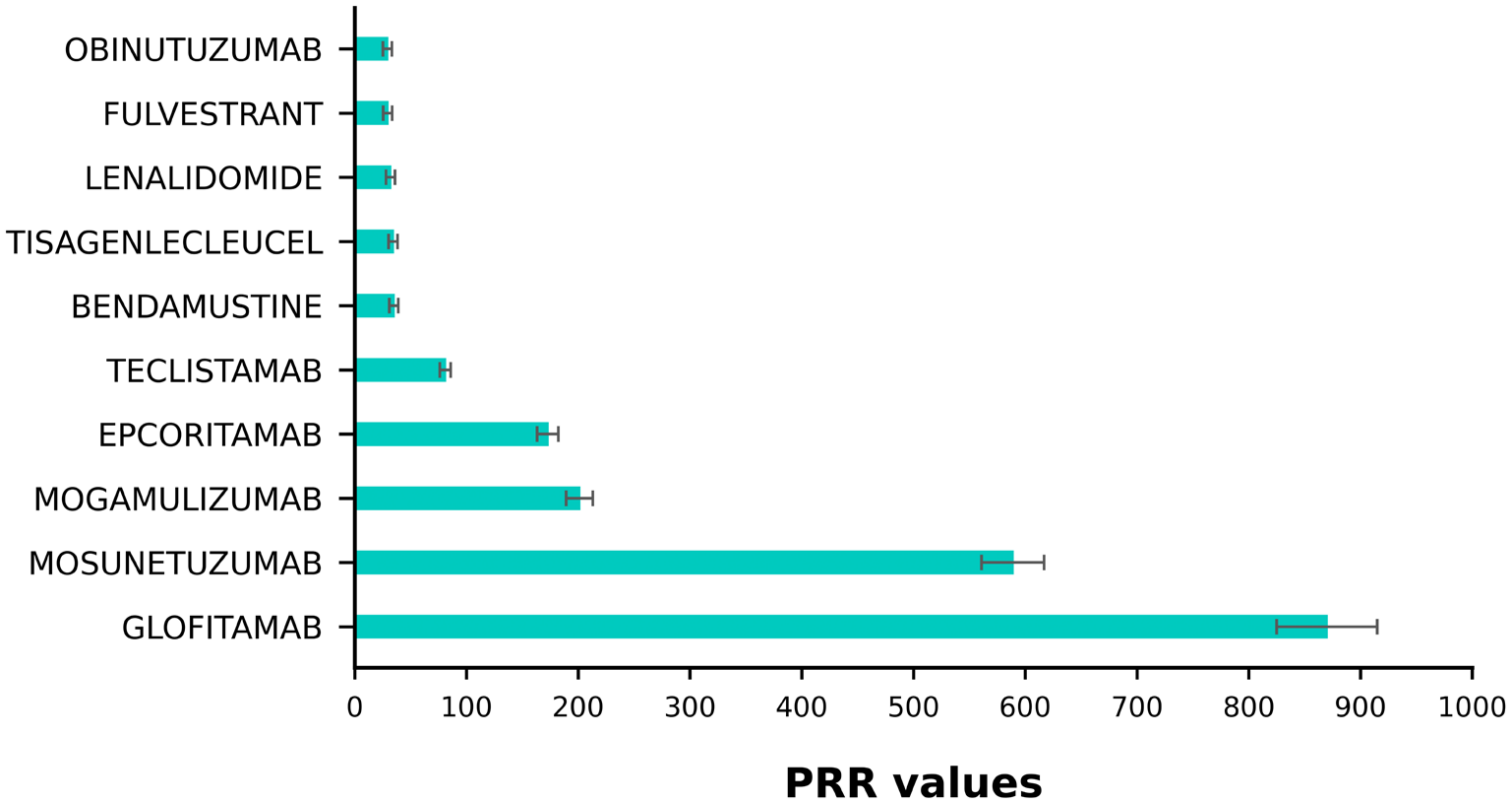
Top 10 drugs ranked by PPR values.

In the PRR model, Glofitamab had the highest PRR value (869.89), followed by Mosunetuzumab (588.87) and Mogamulizumab (201.1). Clearly, this result is consistent with the findings from the ROR model, which enhances the accuracy of the data in this study (Figure 4).

Based on the EBGM model algorithm, we identified several anticancer drugs with high association scores, indicating their potential role in TFR. Through calculations, the drug with the highest EBGM value was Glofitamab (EBGM 846.86), showing the highest association with TFR, followed by Mosunetuzumab (EBGM 580.56) and Mogamulizumab (EBGM 198.27), which also showed high risk. Other important drugs included Epcoritamab (EBGM 171.55) and Teclistamab (EBGM 80.43), all of which had significant EBGM scores.

Finally, we processed the data using the BPCNN algorithm, and the results showed key anticancer drugs significantly associated with TFR. Surprisingly, the top three drugs were consistent with the previous models: Glofitamab had the highest IC value of 9.73, followed by Mosunetuzumab (IC 9.18) and Mogamulizumab (IC 7.63), all of which showed a close association with TFR.

In conclusion, the results from the four models—ROR, PRR, EBGM, and BPCNN—were consistent, which strengthens the robustness of these methods in identifying drugs associated with TFR. Glofitamab, Mosunetuzumab, and Mogamulizumab emerged as the top three drugs, with Mosunetuzumab and Mogamulizumab notably lacking TFR warnings in medication instructions. This finding underscores the importance of our research, suggesting that the clinical community may need to update safety guidelines and more actively monitor TFR in patients receiving these drugs (Table 5).

**TABLE 5:** Top 10 anti‐cancer drugs related to TFR validated by four statistical models.

Drug	ROR	PRR	EBGM	BPCNN
Glofitamab	893.29	869.89	846.86	9.73
Mosunetuzumab	599.63	588.87	580.56	9.18
Mogamulizumab	202.34	201.1	198.27	7.63
Epcoritamab	173.68	172.76	171.55	7.42
Teclistamab	81.2	81	80.43	6.33
Bendamustine	35.07	35.03	34.43	5.11
Tisagenlecleucel	34.35	34.31	34.14	5.09
Fulvestrant	29.48	29.46	29.01	4.86
Obinutuzumab	29.33	29.3	29	4.86
Brentuximab Vedotin	28.18	28.16	27.87	4.8
Lutetium (177LU) Dotatate	23.78	23.76	23.64	4.56
Lenalidomide	32.06	32.04	20.25	4.34
Goserelin	19.14	19.13	19.03	4.25
Sipuleucel‐T	18.56	18.55	18.45	4.21
Fludarabine	16.44	16.44	16.33	4.03

## 4 Discussion

Tumor flare reaction (TFR) represents a clinically significant but under‐recognized adverse event in oncology. Initially described in patients with chronic lymphocytic leukemia (CLL) receiving immunomodulatory drugs (IMiDs), TFR has subsequently been reported across a variety of hematologic and solid tumors, particularly in the era of immune checkpoint inhibitors (ICIs) [4, 5, 8]. Although prior reports have primarily been limited to case series and small cohorts, systematic pharmacovigilance analyses remain scarce. To address this gap, we conducted a large‐scale FAERS‐based study to characterize the clinical features of TFR, identify drug–event associations, and evaluate the broader clinical implications.

In our dataset of 566 FAERS reports from 2004 to 2024, TFR was most frequently associated with hematologic malignancies, especially CLL, but was also observed in diffuse large B‐cell lymphoma, mantle cell lymphoma, and—among solid tumors—prostate cancer. The steady rise in TFR reporting, peaking in 2023, likely reflects both the expanding use of novel immune‐based therapies and increasing clinical awareness of this phenomenon. These trends corroborate previous descriptions that TFR is particularly prevalent in lymphoid malignancies while also extending its relevance to solid tumors [8, 9, 10, 11].

Drug‐level analyses identified 28 anticancer agents associated with TFR through four disproportionality algorithms. Among these, lenalidomide accounted for nearly 38% of all TFR reports, consistent with prior studies describing painful lymphadenopathy, fever, rash, and lymphocytosis as classical manifestations in CLL [4, 5, 8, 12]. Importantly, lenalidomide‐related TFR has been correlated with treatment response, though high‐dose regimens have carried risk for severe or life‐threatening flares [13]. Beyond IMiDs, strong signals were detected for bispecific antibodies and targeted immunotherapies, including glofitamab, mosunetuzumab, and mogamulizumab, which ranked highest across all models. While glofitamab's product labeling explicitly acknowledges tumor flare events, mosunetuzumab has demonstrated approximately 4% incidence of TFR in clinical trials [14], and mogamulizumab, although lacking published incidence data, was also associated with TFR in our dataset. Notably, neither mosunetuzumab nor mogamulizumab consistently include these risks in their current product labeling, underscoring the importance of post‐marketing pharmacovigilance to capture clinically meaningful adverse reactions that may not be fully recognized at the time of approval.

The biological plausibility of these findings is supported by pharmacologic mechanisms. IMiDs enhance T‐ and NK‐cell activation, augment cytokine production, and trigger local inflammatory responses, accounting for their strong association with TFR. ICIs such as nivolumab may induce transient tumor swelling due to T‐cell infiltration, potentially mimicking disease progression [3, 7, 8]. Bispecific antibodies, by directly bridging effector T cells to malignant B cells, induce rapid and pronounced immune activation, which may explain the exceptionally high disproportionality scores for glofitamab and mosunetuzumab [14, 15]. These mechanistic distinctions are clinically relevant, as TFR is often symptomatic and acute, whereas pseudoprogression—another unconventional response pattern—is generally radiographic, asymptomatic, and confirmed only with serial imaging [15, 16].

Clinically, our findings support three practical recommendations. First, in patients with early apparent progression after treatment initiation, TFR should be carefully considered, particularly when systemic symptoms (fever, rash, painful lymphadenopathy) accompany radiologic changes. Second, patients who remain clinically stable may be managed with supportive care (analgesics, corticosteroids when indicated) and short‐interval reassessments rather than discontinuing therapy prematurely [8, 17]. Third, multidisciplinary evaluation, including radiology review, is essential to distinguish TFR from pseudoprogression or true progression. In immunotherapy settings, the use of iRECIST provides a standardized framework for response assessment when atypical radiographic patterns are observed [18].

Compared with existing literature, our study not only confirms well‐documented associations such as lenalidomide‐induced TFR in CLL but also highlights emerging concerns for novel targeted therapies, including glofitamab, mosunetuzumab, and mogamulizumab. These signals, consistently detected across all algorithms, warrant close clinical monitoring and consideration in regulatory labeling.

The strengths of this study include the use of a large pharmacovigilance database spanning two decades, application of multiple complementary algorithms, and a narrow MedDRA‐based definition of TFR. Limitations, however, must be acknowledged: FAERS data are subject to under‐reporting, missing or incomplete clinical information, and the absence of denominators, precluding incidence estimation and causal inference [19]. Thus, our results should be interpreted as indicators of disproportionate reporting rather than definitive risk estimates.

In conclusion, this FAERS‐based analysis demonstrates that TFR is disproportionately reported across diverse anticancer therapies, with both established and novel agents implicated. Early recognition and differentiation from pseudoprogression are critical to avoid unnecessary treatment discontinuation. Our findings serve as a clinical alert, emphasizing the need for heightened vigilance, improved labeling, and prospective studies to refine diagnostic criteria and management strategies for TFR in the modern oncology landscape.

## Author Contributions

Wei Wang, Jiayuan Cui, and Yuxuan Bo drafted the manuscript. Yuxuan Wang, Zhipeng Sun, and Xin Guan were significant contributors to the revision of the manuscript, Ning Zhang and Baoan Hong conceived the present study. All authors read and approved the final manuscript.

## Funding

This study was supported by the Beijing Hospitals Authority Youth Programme (QML20231105), Beijing Bethune Charitable Foundation (mnzl202003), Beijing Anzhen Hospital High‐Level Research Funding (2024AZC3001), Beijing Anzhen Hospital Science and Technology Development Funding (AZYZR202304), Beijing Anzhen Hospital High‐Level Research Special Program (Incubation Program) (2025AZB6018).

## Conflicts of Interest

The authors declare no conflicts of interest.

## Supporting information


**Table S1:** All anti‐cancer drugs related with TFR validated by all four statistic models.

## Data Availability

All the data can be retrieved form the FAERS Publish Dashboard (https://www.fda.gov/drugs/questions‐and‐answers‐fdas‐adverse‐event‐reporting‐system‐faers/fdaadverse‐event‐reporting‐system‐faers‐public‐dashboard).
