# Supplementary material for: Evaluation of Anticancer Therapy‐Related Tumor Flare Reaction: Insights From Food and Drug Administration's Adverse Event Reporting System Dataset
Source: Cancer Med. 2026 Mar 9;15(3):e71660. doi: 10.1002/cam4.71660 (PMC12971376; doi:10.1002/cam4.71660)
Supplement: Supplementary file 1 — Table S1: All anti‐cancer drugs related with TFR validated by all four statistic models. [file CAM4-15-e71660-s001.docx]

Supplementary Materials

Supplementary Table: All anti-cancer drugs related with TFR validated by all four statistic models

| Drug | ROR | PRR | EBGM | BPCNN |
| --- | --- | --- | --- | --- |
| GLOFITAMAB | 893.29 | 869.89 | 846.86 | 9.73 |
| MOSUNETUZUMAB | 599.63 | 588.87 | 580.56 | 9.18 |
| MOGAMULIZUMAB | 202.34 | 201.1 | 198.27 | 7.63 |
| EPCORITAMAB | 173.68 | 172.76 | 171.55 | 7.42 |
| TECLISTAMAB | 81.2 | 81 | 80.43 | 6.33 |
| BENDAMUSTINE | 35.07 | 35.03 | 34.43 | 5.11 |
| TISAGENLECLEUCEL | 34.35 | 34.31 | 34.14 | 5.09 |
| FULVESTRANT | 29.48 | 29.46 | 29.01 | 4.86 |
| OBINUTUZUMAB | 29.33 | 29.3 | 29 | 4.86 |
| BRENTUXIMAB VEDOTIN | 28.18 | 28.16 | 27.87 | 4.8 |
| LUTETIUM (177LU) DOTATATE | 23.78 | 23.76 | 23.64 | 4.56 |
| LENALIDOMIDE | 32.06 | 32.04 | 20.25 | 4.34 |
| GOSERELIN | 19.14 | 19.13 | 19.03 | 4.25 |
| SIPULEUCEL-T | 18.56 | 18.55 | 18.45 | 4.21 |
| FLUDARABINE | 16.44 | 16.44 | 16.33 | 4.03 |
| RITUXIMAB | 15.8 | 15.8 | 14.67 | 3.88 |
| LUTETIUM (177LU) VIPIVOTIDE TETRAXETAN | 13.58 | 13.58 | 13.51 | 3.76 |
| ANASTROZOLE | 13.61 | 13.61 | 13.48 | 3.75 |
| RIBOCICLIB | 11.97 | 11.97 | 11.85 | 3.57 |
| THALIDOMIDE | 11.89 | 11.89 | 11.71 | 3.55 |
| NIVOLUMAB | 11.1 | 11.1 | 10.73 | 3.42 |
| DURVALUMAB | 8.95 | 8.94 | 8.9 | 3.15 |
| PEMETREXED | 7.92 | 7.92 | 7.89 | 2.98 |
| IBRUTINIB | 6.55 | 6.55 | 6.42 | 2.68 |
| DOXORUBICIN | 6.14 | 6.14 | 6.08 | 2.6 |
| OSIMERTINIB | 6.01 | 6.01 | 5.98 | 2.58 |
| PEMBROLIZUMAB | 5.71 | 5.71 | 5.64 | 2.5 |
| CYCLOPHOSPHAMIDE | 5.55 | 5.55 | 5.5 | 2.46 |
